# State of knowledge on ammonia handling by the kidney

**DOI:** 10.1007/s00424-024-02940-1

**Published:** 2024-03-07

**Authors:** Soline Bourgeois, Pascal Houillier

**Affiliations:** 1 Institut of Physiology, University of Zurich, Zurich, Switzerland; 2 Centre de Recherche Des Cordeliers, INSERM, Sorbonne Université, Université Paris Cité, Paris, France; 3 Centre National de La Recherche Scientifique (CNRS), EMR 8228, Paris, France

**Keywords:** Renal, Ammonia transport, Proximal tubule, Thick ascending limb, Collecting duct, Acid–base homeostasis

## Abstract

The disposal of ammonia, the main proton buffer in the urine, is important for acid–base homeostasis. Renal ammonia excretion is the predominant contributor to renal net acid excretion, both under basal condition and in response to acidosis. New insights into the mechanisms of renal ammonia production and transport have been gained in the past decades. Ammonia is the only urinary solute known to be produced in the kidney and selectively transported through the different parts of the nephron. Both molecular forms of total ammonia, NH_3_ and NH_4_^+^, are transported by specific proteins. Proximal tubular ammoniagenesis and the activity of these transport processes determine the eventual fate of total ammonia produced and excreted by the kidney. In this review, we summarized the state of the art of ammonia handling by the kidney and highlighted the newest processes described in the last decade.

## Introduction

Respiratory (CO_2_ production) and metabolic processes (food intake and cellular metabolism) continuously add acid to the body, challenging acid–base balance. The adjustment of extracellular pH in a narrow range from 7.36 to 7.44 is critical for cellular and organ performances. For this reason, disorders of acid–base homeostasis are associated with the worsening of kidney function and a higher morbidity and mortality in patients with chronic kidney disease (CKD) [3]. Moreover, it has been shown in the general population under physiological conditions that polymorphisms in genes involved in renal ammonia transport and metabolism are associated with values of urine pH and may contribute to the pathogenesis of kidney diseases associated with acid retention [22]. Extracellular pH affects bone density and stability, and mild chronic metabolic acidosis has been suspected to contribute to osteopenia and osteoporosis [1, 61]. Rickets and osteomalacia are often observed in patients with inborn syndromes of renal tubular acidosis. Extracellular acidosis affects skeletal muscle metabolism and induces a catabolic state [3].

The kidney is one of the key organs controlling and maintaining a normal systemic acid–base status, through three mechanisms: the reabsorption of filtered bicarbonate, the de novo generation of ammonium (NH_4_^+^) and bicarbonate, and the excretion of net acid, mostly under the form of ammonium. Amino acid metabolism in the standard non-vegan or non-vegetarian Western diet is an acidifying diet rich in animal protein and results in endogenous acid production of around 0.8 to 1.0 mEq/kg/day [12]. To replenish the body’s bicarbonate content, the proximal tubule produces ammonium, which is secreted by the collecting duct (CD) in the form of ammonia and protons to form ammonium in the urine. Dysfunction of either of these two steps leads to proximal (pRTA) and distal (dRTA) renal tubular acidosis, respectively. Ammonia excreted in the final urine as NH_4_^+^ leads to the formation of a new equimolar bicarbonate, while ammonia returning to the systemic circulation via the renal vein is metabolized by the liver to urea and glutamine [57].

## Biochemistry of ammonia

Under biological conditions, total ammonia (t-ammonia) exists both as ammonia (NH_3_) and its protonated form ammonium (NH_4_^+^). The relative concentration of each of these forms in bodily fluids is driven by the buffering reaction NH_3_ + H^+^– > NH_4_^+^. The abundance of t-ammonia makes NH_3_ the main H^+^ buffer in the urine.

In the body, the pKa of this reaction is around 9.2. Thus, under physiological conditions, as the biological fluids exhibit a pH below the pKa, most of the t-ammonia exists as NH_4_^+^; for example, NH_3_ accounts for a mere 1% of the t-ammonia in serum at pH 7.4 and is only present in trace amounts in urine where pH is generally below 6 [49].

## Overview of ammonia transport in the nephron

Four decades ago, micropuncture and in vivo microperfusion studies assessed the fate of ammonia in the kidney. T-ammonia is produced and secreted into the luminal fluid at the level of the proximal tubule [19, 20, 45, 47]. In the loop of Henle, and more predominantly, in the medullary thick ascending limb, t-ammonia is reabsorbed and concentrated by countercurrent multiplication, inducing an interstitial ammonia concentration gradient that increases from the cortex to the outer medulla and then again to the inner medulla [91, 106].

**Fig. 1:**
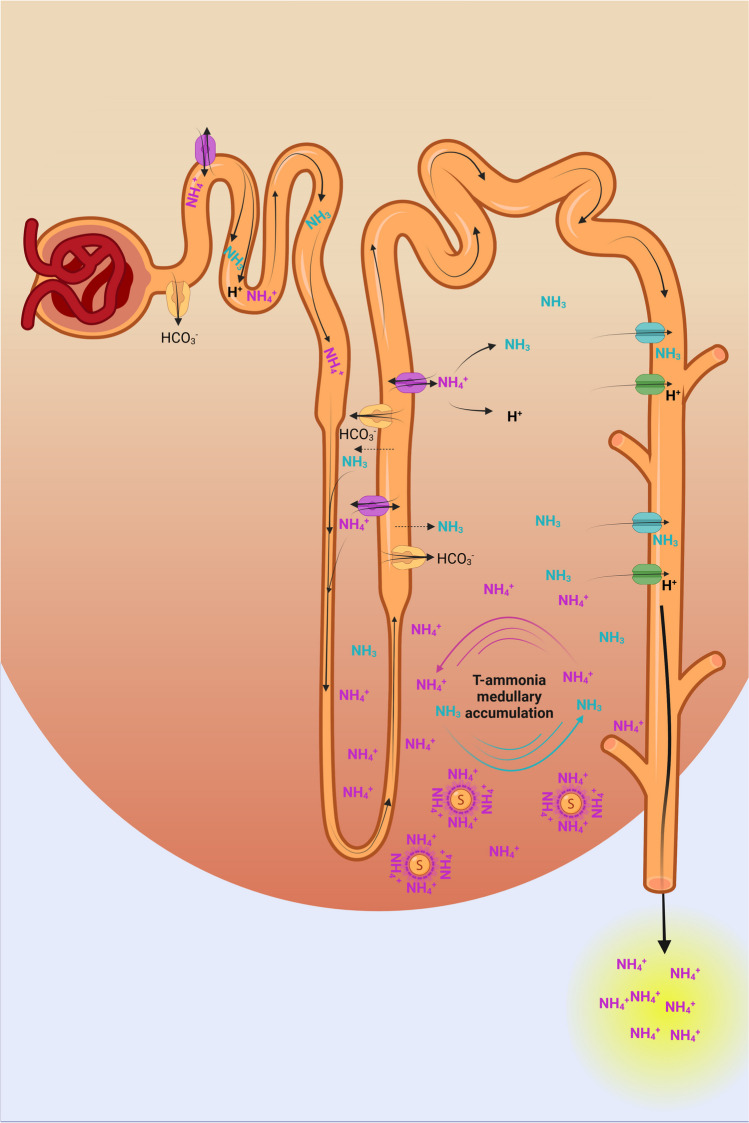
Urinary t-ammonia generation and transport in the nephron. NH_3_/NH_4_^+^ (t-ammonia) is produced essentially from glutamine metabolism into the proximal tubular (PT) cells. T-ammonia is secreted into the luminal fluid at the level of PT. In the loop of Henle, and more predominantly, in the medullary thick ascending limb (TAP), t-ammonia is reabsorbed and concentrated by counter-current multiplication which induces an interstitial ammonia concentration gradient that increases from cortex to outer medulla to inner medulla. There, sulfatides (S) accumulate in kidney interstitium where they can reversibly bind NH_4_^+^. Finally, ammonia (NH_3_) is secreted by the adjacent collecting duct, to reach final urinary t-ammonia excretion rate. Created with BioRender.com

Finally, ammonia is secreted by the adjacent collecting duct to reach final urinary t-ammonia excretion rate [102]. Production, transport, and final excretion of t-ammonia along the nephron increase in metabolic acidosis in the rat kidney (Fig. 1) [100, 102].

## Ammonia metabolism and reabsorption in the proximal part of the nephron

### Renal glutamine transport and ammonia production

Ammonia is a highly toxic compound, particularly for central nervous system cells. Concentration in plasma is low, in the micromolar range (https://www.merckmanuals.com/professional/resources/normal-laboratory-values/normal-laboratory-values). Unlike other urine constituents, most of the t-ammonia found in urine does not stem from plasma content filtered by the glomerulus but is rather produced within the nephrons by the proximal tubular cell. The rate of ammoniagenesis is highly dependent on acid–base status. In the context of a Western diet, the absence or deficiency of one of the protagonists of ammonia production, metabolism, and transport in the proximal tubule can lead to the retention of non-volatile acids in the body and the development of metabolic acidosis.

**Fig. 2:**
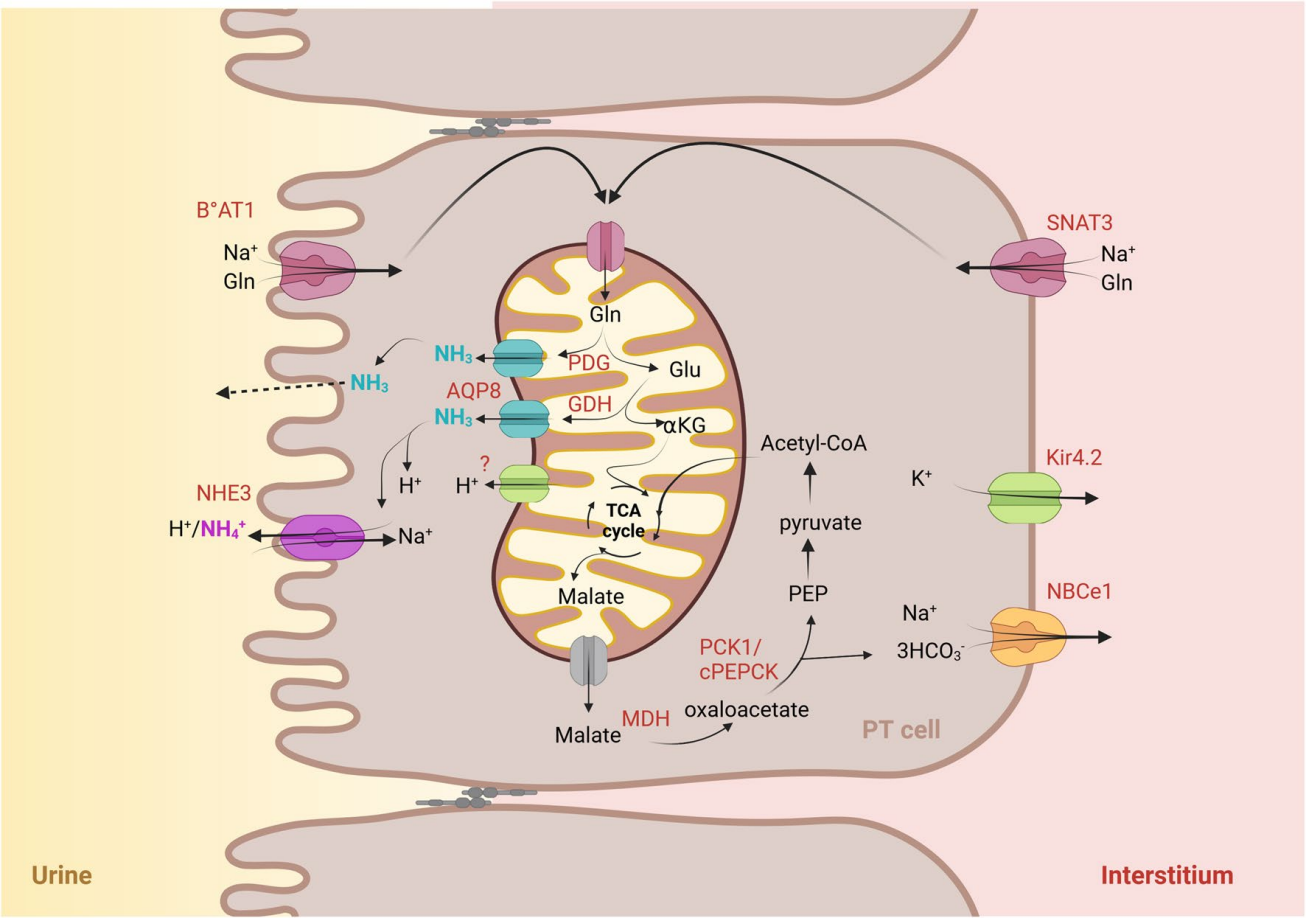
T-ammonia generation and transport in the proximal tubule (PT) cells. Glutamine is carried into the PT epithelial cells by the amino acid cotransporters B°AT1 and SNAT3. In the PT cells, glutamine is taken up by the mitochondria and processed to one α-ketoglutarate and 2 NH_4_^+^ molecules by the action of phosphate-dependent glutaminase (PDG) and glutamate dehydrogenase (GDH). α-Ketoglutarate metabolized in the tricarboxylic acid (TCA) cycle results in the generation of malate which is secreted into the cell cytoplasm through the mitochondrial membrane. In the cytoplasm, malate metabolism results in the generation of one bicarbonate molecule after the action of malate dehydrogenase and cytoplasmic phosphoenolpyruvate carboxykinase (c-PEPCK or PCK-1) to form phosphoenolpyruvate (PEP). PEP can then be metabolized to form pyruvate and undergo again TCA cycle or used by the proximal cell for gluconeogenesis. The newly formed NH_4_^+^ is leaving the mitochondria via the aquaporin isoform 8 (AQP8). The t-ammonia (NH_3_/NH_4_^+^) formed in PT cells is secreted into the tubular lumen by diffusion across the apical membrane and by active transport, mainly via the Na/H exchanger NHE3 by proton substitution or via a barium-sensitive K channel. The bicarbonate (HCO_3_^−^) formed is transported across the basolateral plasma membrane by the Na-bicarbonate/carbonate (HCO_3_^c−^/CO_3_^2−^) cotransporter NBCe1 and transferred to the bloodstream. The activity of the Kir4.2 potassium channel hyperpolarizes the plasma membrane to enable activation of carbonate transport by the NBCe1 transporter. This process lowers the intracellular pH required for the proper functioning of ammoniagenic enzymes and ammonium transporters. Created with BioRender.com

NH_4_^+^ is generated mainly by the metabolism of amino acids such as glutamine, which results in the equimolar formation of bicarbonate transported in the blood while ammonium is secreted in the pro-urine (Fig. 2). Glutamine, which is a small molecule, is entirely filtered by the glomerulus and taken up by the proximal tubule [100]. In the proximal tubule, glutamine is transported into epithelial cells at least in part by the amino acid cotransporters B°AT1 (SLC6A19) and SNAT3 (SLC38A3) at the apical and basolateral poles of the cell, respectively [27, 84]. Glutamine is then taken up by the mitochondria via yet unknown transporters. In mitochondria, glutamine is converted into one α-ketoglutarate and 2 NH_4_^+^ molecules by the action of phosphate-dependent glutaminase (PDG) and glutamate dehydrogenase (GDH). α-Ketoglutarate is metabolized as part of the tricarboxylic acid (TCA) cycle, generating malate which is metabolized to form phosphoenolpyruvate (PEP) by the mitochondrial phosphoenolpyruvate carboxykinase (m-PEPCK or PCK2) or secreted into the cell cytoplasm through the mitochondrial membrane. In the cytoplasm, malate metabolism leads to the production of a bicarbonate molecule after the action of malate dehydrogenase and cytoplasmic phosphoenolpyruvate carboxykinase (c-PEPCK or PCK-1) to form PEP ([109], for review [56, 123]). The importance and rate-limiting effect of PCK1 has been highlighted recently by the study of PCK1 knockout mice. Even though PCK1 acts in the cytoplasm of proximal tubular cells, its disruption leads to severe dysfunction of the mitochondria, an inability of the mice to fight against an acid load and the development of metabolic acidosis due to a decrease of ammoniagenesis [109]. PCK1 has also been associated with diabetic nephropathy in humans [126]. PEP can then be metabolized to form pyruvate and undergo the TCA cycle again or be used by the proximal cell for gluconeogenesis [123]. T-ammonia formed during the metabolism of glutamine leaves the mitochondria for the cell cytoplasm. A member of aquaporin family, AQP8, has been first localized in the mitochondrial membrane of rat kidney and suggested as NH_3_ channel involved in ammonia diffusion from the mitochondria to the cytoplasm of proximal tubular cells [33, 83]. Indeed, in addition to its ability to transport H_2_O, mouse, rat, or human AQP8 when heterologously expressed in cell lines has been shown to allow NH_3_ diffusion [58, 77, 96]. In the human renal proximal cell line, HK-2, which expresses AQP8 in its inner mitochondrial membrane, the knockdown of AQP8 decreased ammonia secretion by these cells. The authors also demonstrated that mitochondrial AQP8 expression increased in acid-loaded rats during chronic metabolic acidosis, supporting a role of AQP8 in the kidney’s response to an acid challenge [83]. These data support a role for AQP8 in native proximal tubule mitochondrial T-ammonia transport. However, the physiological relevance of AQP8 role in ammonia handling by the proximal tubule is still questioned by the mild phenotype of AQP8 knockout mice [128]. Indeed, AQP8 knockout mice, which had no defect under baseline conditions, showed increased renal ammonia excretion during experimental metabolic acidosis created by chronic NH_4_Cl challenge, and no change in blood pH and blood pCO_2_ compared with wild-type mice [128]. Further research is therefore needed to resolve these contradictions and assess a possible compensatory effect of other aquaporins/transporters in t-ammonia export out of the proximal cell mitochondria [82, 83].

### Ammonia transport in the proximal tubule

Ammonium formed in proximal tubular cells is secreted into the tubular lumen by diffusion across the apical membrane and by active transport mainly through the Na/H exchanger NHE3 (SLC9A3) by protons substitution, or through a barium-sensitive K^+^ channel [48]. NH_4_^+^ and K^+^ ions have almost identical biophysical characteristics, which allows NH_4_^+^ ions to be transported by theoretically all K^+^ transporters in epithelial cells [43, 121]. In parallel, the bicarbonate formed during malate metabolism is transported across the basolateral plasma membrane mainly by the Na-bicarbonate/carbonate (HCO_3_^−^) cotransporter NBCe1 (SLC4A4) and transferred into the bloodstream with a stoichiometry of 1 Na^+^ for 3HCO_3_^−^ [10, 72]. Recently, it has been reported by Lee and colleagues in a mechanistic study that NBCe1 transports the main blood H^+^ buffer under its CO_3_^2−^ form [72]. So far, no other technical approach, such as structural studies, has been able to assess this crucial question. The recent crystal structure of NBCe1 at 3.9 Å could not determine with precision the exact substrate for NBCe1 coupled to Na^+^ [60]. Further research is needed to determine the stoichiometry of this transport, as the 1:3 stoichiometry cannot be maintained with the CO_3_^2−^ form [60, 69, 72]. The picture of ammonia handling by the proximal tubule has recently been better extended by the demonstration of the role of the potassium channel Kir4.2 as a powerful determinant of ammoniagenesis as well as of ammonia and bicarbonate transport by the proximal tubule [4]. In male mice, deletion of Kir4.2 causes metabolic acidosis via depolarization of the basolateral plasma membrane and impairment of bicarbonate handling by the NBCe1 transporter. This process increases intracellular pH and, consequently, alters the expression of ammoniagenic enzymes and ammonium transporters (Fig. 2) [4].

## Transport in the thick ascending limb and concentration in renal medulla

**Fig. 3:**
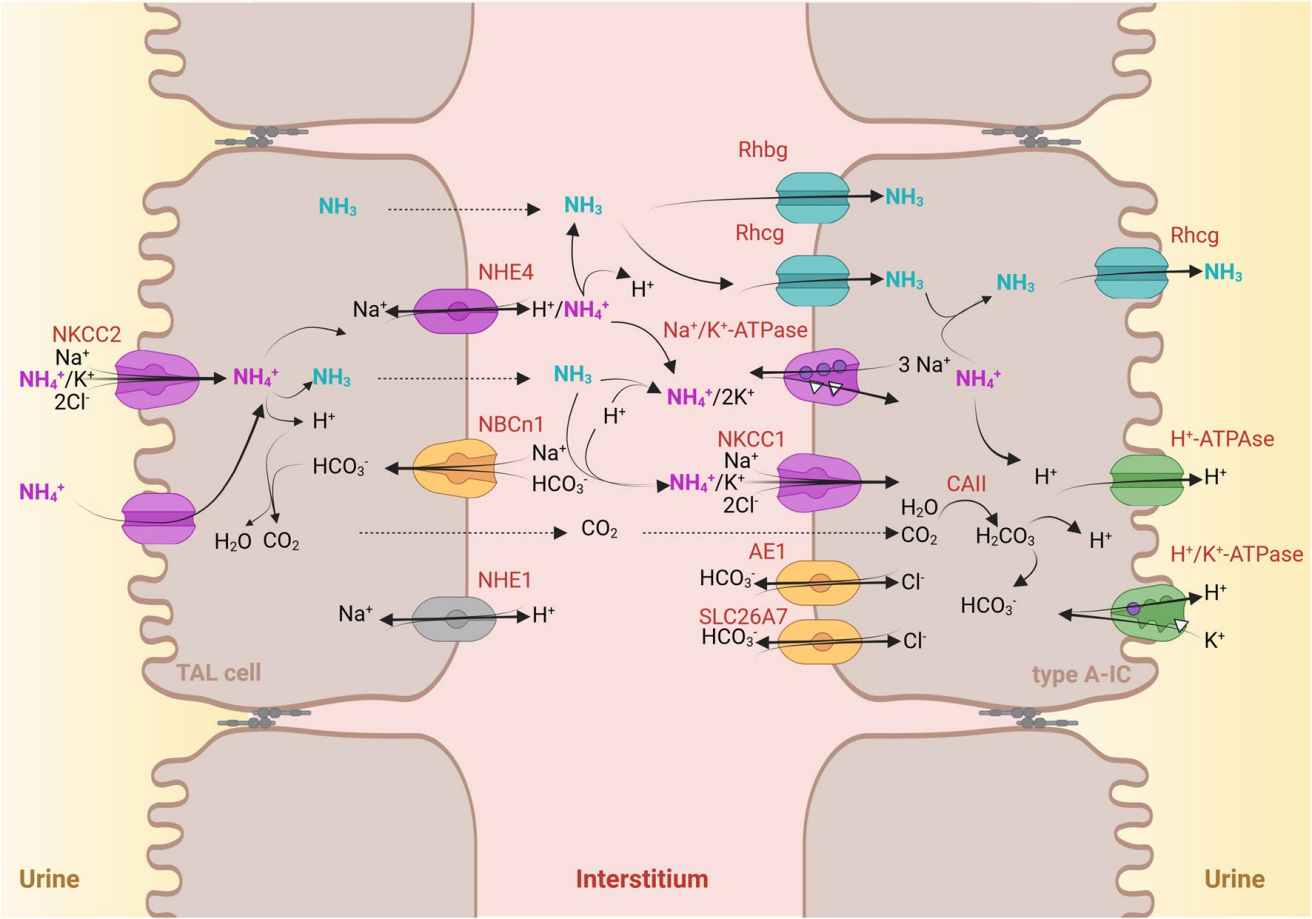
T-ammonia reabsorption in the thick ascending limb (TAL) cells and secretion in the collecting duct cells work in concert to facilitate NH_4_^+^ excretion in the urine. In the TAL, NH_4_^+^ is reabsorbed essentially by the Na^+^-K^+^-2Cl^−^ cotransporter isoform 2, NKCC2 and to a lesser extent, a barium sensitive K^+^ channel. Intracellular t-ammonia leaves the cell at the basolateral side via a direct NH_4_^+^ transport, the Na^+^/(H^+^/NH_4_^+^) exchanger NHE4, and an indirect process of NH_3_ transport by bicarbonate buffering involving the electroneutral cotransporter Na^+^-HCO_3_^−^ NBCn1. In, the type A IC, t-ammonia is taken up as NH_4_^+^ by the Na^+^/(K^+^/NH_4_^+^)-ATPase or the cotransporter Na^+^-K^+^-2Cl^−^ isoform 1 NKCC1 or NH_3_ by the Rhesus (Rh) glycoproteins Rh C glycoprotein (Rhcg) or Rh B glycoprotein (Rhbg) thanks to the cortico-papillary gradient of NH_3_/NH_4_^+^. Within the cell cytoplasm, carbonic anhydrase II (CAII) facilitates the basolateral efflux of HCO_3_^−^ by activating the Cl^−^/HCO_3_^−^ exchangers AE1 and SLC26A7 which allows the generation of H^+^ in the cytosol and enable parallel H^+^ and NH_3_ secretions across the apical plasma membrane by, respectively, Rhcg and primarily, the multimeric protein H^+^-ATPase or the electroneutral H^+^,K^+^-ATPase. Created with BioRender.com

The next site where significant transport of t-ammonia takes place is the loop of Henle and where most t-ammonia is reabsorbed in the thick ascending limb (TAL). Part of the reabsorbed t-ammonia is passively secreted into the thin descending limb [36], a phenomenon that plays a role in countercurrent multiplication and creates a cortico-papillary concentration gradient of NH_3_/NH_4_^+^ (Fig. 1) that allows NH_3_/NH_4_^+^ capture and secretion into the adjacent collecting duct (Fig. 3) [13, 43]. The magnitude of the concentration gradient increases with acidosis and water deprivation and drastically decreases during alkali load and furosemide infusion [91, 93]. The inability to create or maintain a cortico-papillary gradient of NH_3_/NH_4_^+^ can presumably be responsible for the progressive decrease in ammonium excretion that characterizes chronic kidney disease [18]. However in conditions where this gradient is disrupted, the body can still compensate for the lack of ammonia reabsorption in the TAL by increasing the urine output and thus ammonium supply to collecting duct cells, as has been observed in NHE4 and NBCn1 knockout mice [16, 89].

### Luminal uptake

In the TAL, most of NH_4_^+^ reabsorption occurs transcellularly. The main transporter enabling apical uptake of ammonia from the tubular lumen is the Na^+^-K^+^-2Cl^−^ cotransporter isoform 2, NKCC2 (SLC12A1). Inhibition of NKCC2 by furosemide suppresses NH_4_^+^ reabsorption in the ex vivo microperfused rat medullary TAL [45]. The NH_4_^+^ ion enters the cells by substituting for K^+^ (Fig. 3) [39, 43, 66]. As the NH_4_^+^ and the K^+^ ions compete at the same protein site, modulations of luminal K^+^ concentration also modify TAL NH_4_^+^ reabsorption. Thus, a lower extracellular potassium concentration increases ammonia reabsorption, and a higher potassium concentration reduces it [40, 41]. NKCC2 protein activity is modulated mainly by phosphorylation and trafficking [42, 86]. However, detailed knowledge of its role in the TAL response to ammonia handling remains incomplete. In addition to NKCC2, the activity of a luminal barium sensitive K^+^ channel has been documented in the ex vivo microperfused TAL [120]. The physiological importance of this channel in ammonia transport appears to be limited as the pharmacological inhibition of NKCC2 by furosemide almost abolishes ammonia reabsorption in the ex vivo microperfused TAL.

### Basolateral exit

To complete ammonia reabsorption, intracellular ammonia must be transported across the basolateral membrane of TAL cells. Ex vivo microperfusion studies on TAL have demonstrated that 1 mM amiloride or 50 µM EIPA, an amiloride analogue, in the peritubular fluid drastically reduced ammonia reabsorption [44]. More than a decade later, two different transporters, the electroneutral Na^+^-HCO3^−^ cotransporter NBCn1(SLC4A7) and the Na^+^/H^+^ exchanger NHE4 (SLC9A4) operating as a Na^+^/NH_4_^+^ exchanger, were shown to be involved in this final step of ammonia transport across the TAL (for review [59]).

NBCn1, first cloned from the rat smooth muscle cell [28], has been functionally identified in the basolateral membrane of the rat medullary thick ascending limb cells and localized in the rat and mouse TAL and mouse medullary collecting duct cells [8, 15, 111]. Being electroneutral, NBCn1 acts as a base loader, drawing sodium and bicarbonate into the cell. There, bicarbonate buffers a hydrogen ion from NH_4_^+^, releasing NH_3_ that then diffuses across the basolateral membrane [89]. The abundance of NBCn1 protein in the kidney and its activity in the ex vivo microperfused medullary TAL cells increase significantly during chronic metabolic acidosis induced by NH_4_Cl loading in rats and mice [71, 88, 89]. However, although its expression is controlled by acid–base status, the role of NBCn1 in ammonium reabsorption by the TAL and ammonium excretion in urine has been unclear until the recent phenotyping of NBCn1 knockout mice [89]. When exposed to NH_4_Cl in drinking water, NBCn1 knockout mice showed an abnormal adaptation to the acid load compared to wild-type mice. In TAL cells, net ammonia reabsorption was significantly reduced in NBCn1 knockout mice loaded with acid for 1 day [89]. Basolateral Na/H activity was maximally elevated under control and plateaued under acid loading compared with wild-type TAL [89]. The basolateral membrane of the medullary TAL cells expresses two distinct Na^+^/H^+^ exchangers: the ubiquitous NHE1 isoform and the TAL-specific NHE4 isoform [26]. NHE4 is highly resistant to amiloride derivatives: in purified basolateral vesicles, the IC_50_ value for ethyl isopropyl amiloride (EIPA) has been calculated to be 2.5 µM, contrasting with the high sensitivity of NHE1 (IC_50_ = 11 nM) [26]. Furthermore, in PS120 fibroblasts transfected with NHE4 isoform, 1 mM amiloride inhibits by 80% the NHE4-dependent Na/H activity [9]. Of note, when Olsen et al. used 1 mM amiloride to inhibit the basolateral Na/H activities, they were unable to distinguish between NHE1 and NHE4 activities [9, 16, 24]. NHE1 and NHE4 also differ in their respective sensitivity to intracellular pH (pHi), NHE4 being active at a lower pHi than NHE1 (pK values 6.21 and 6.75, respectively) [26, 90]. Medullary TAL cells have an intracellular pH around 6.9–7 when studied in the absence of extracellular ammonium, and NHE4 is probably almost inactive. When medullary TAL cells absorb ammonium, their intracellular pH reduces to about 6.5–6.6 [120], which is expected to activate NHE4.

NHE4 mRNA expression and protein activity are increased during metabolic acidosis in mice and rats, respectively [16]. The direct demonstration that NHE4 is important for the basolateral exit of NH_4_^+^ has been provided by the study of the phenotype of NHE4 knockout mice [16]. On a control diet, NHE4 knockout mice exhibit a compensated, hyperchloremic metabolic acidosis with elevated excretion of ammonium in the urine compared to their littermate wild type [16]. However, the renal net acid excretion is not different from that of normal mice, revealing an inability to excrete more acid as expected during metabolic acidosis. It is noteworthy that the urinary pH of NHE4 knockout mice was lower than that of wild-type mice, suggesting that (1) the absence of NHE4 does not impair hydrogen ion secretion in the collecting duct and (2) the NHE4 knockout mice may have developed a compensatory increase in diffusion gradient of NH_3_ by the adjacent collecting duct to support final t-ammonia excretion, likely caused by an increased activity of NH_3_ channels such as Rhcg or Rhbg and the multimeric protein H-ATPase of the A-IC. In addition, the lower urinary pH could facilitate ammonia diffusion across the collecting duct epithelium. However, these hypotheses have not been investigated in NHE4 knockout mice. When subjected to chronic dietary acid loading, NHE4 knockout mice show worsening metabolic acidosis, whereas wild-type mice recover from acidosis [16]. The most striking difference between wild-type and NHE4 knockout mice is the latter’s inability to properly increase urinary ammonium excretion (Fig. 3). Good et al. emphasized the importance of medullary ammonia accumulation, and hence ammonia uptake in the loop of Henle, as the main determinant of ammonia secretion in the internal medullary collecting duct in vivo [46]. In agreement, NHE4 knockout mice as NBCn1 knockout mice have an impaired ability to build their cortico-papillary concentration gradient of NH_3_/NH_4_^+^ (Fig. 3). This is probably because medullary TAL cells reabsorb much less ammonia in the absence of NHE4 than in its presence, although NBCn1 could partially compensate.

## Renal interstitium: role of sulfatides in ammonium handling

Sulfatides are a subclass of anionic glycosphingolipids, which accumulate in mammalian kidney with a higher concentration in distal medullary nephron [78]. Disturbances in renal sulfatide content or metabolism have been associated with a large panel of kidney and metabolic diseases [80]. For instance, abnormalities in the glycosphingolipid sulfatide content and activity have been reported in a mouse model of polycystic kidney disease [30], a condition known to progress towards the development of metabolic acidosis in rats [21]. Using molecular genetics and cellular and physiological approaches in a mouse model of disrupted sulfatide synthesis in the kidney, Stettner et al. demonstrated that sulfatides are required for renal excretion of ammonia. When subjected to acidic loading, mice deficient in renal sulfatide synthesis has impaired ammonia excretion with lower ammonia accumulation in the papilla and develop chronic hyperchloremic metabolic acidosis. Sulfatides are highly charged anion glycosphingolipids that may reversibly bind NH_4_^+^ in the renal interstitium (Fig. 1) [105]. Sulfatides, probably through their ability to reversibly bind interstitial NH_4_^+^, are also a key player in renal ammonia handling, urinary acidification, and acid–base homeostasis. However, the precise mechanisms of the disruption of the cortico-papillary ammonia/ammonium gradient remain unknown and need further research to precisely decipher the role of sulfatides in this process.

## Fine-tuning of acid excretion in the distal nephron and the collecting duct

### The ultimate fine-tuning of renal acid excretion takes place in the distal part of the nephron

Intercalated cells, which are highly specialized in transepithelial acid and base transport, are present in the late distal convoluted tubule (DCT2), connecting tubule (CNT), and collecting duct (CD) from the cortex (CCD) to the inner medulla (IMCD) [65]. Roughly, 40% of the cells in CNT, CCD, and outer medulla (OMCD) are IC. IC gradually disappear from the proximal to the last part of IMCD to be virtually absent in the papilla [2]. At least, two intercalated cells subtypes, type A and non-type A, can be recognized and differ by lateralization of the multimeric plasma membrane H^+^-ATPase and expression of various proteins involved in acid and base transport [65]. Type A intercalated cells express the vacuolar H^+^-ATPase at the apical membrane and the Cl^−^/HCO_3_^−^ exchanger AE1 at the basolateral membrane. They are typically involved in proton and ammonium secretion and are expressed from DCT2 to IMCD. The non-type A intercalated cells express the vacuolar ATPase either at the basolateral membrane (also called type B) or at both basolateral and apical membranes (also called non-type A non-type B), and the Cl^−^/HCO_3_^−^ pendrin exchanger at the luminal membrane. Non-type A intercalated cells are mainly expressed in cortical segments, from DCT2 to CCD [65]. Initial studies on isolated and ex vivo microperfused rabbit and rat collecting ducts have shown that the transport of ammonium is predominant in OMCD and IMCD, where only type A intercalated cells are present, and this is tightly regulated by acid–base status (Fig. 3) [95]. In metabolic acidosis, a marked increase in ammonia secretion is also found in the CNT and CCD [67].

The molecular mechanisms of t-ammonia secretion were poorly understood until recent years. The importance of acid secretion in the collecting duct to overall systemic acid–base balance is highlighted by several rare inherited disorders affecting acid–base transport proteins from the collecting duct. Pathogenic variants in the B1 and a4 subunits of H^+^-ATPase (*ATP6V1B1*, *ATP6V0A4*) and in AE1 (*SLC4A1*) have been identified as the cause of distal renal tubular acidosis (dRTA) almost two decades ago [62]. More recently mutations of the transcription factor FoXI1 and of the scaffolding protein WDR72, which are also expressed in the collected duct, have been causally linked to dRTA in humans [34, 94].

### Basolateral uptake

Na^+^,K^+^-ATPase was the first discovered plasma membrane protein complex that was directly involved in NH_4_^+^ uptake by the collecting duct cells [70]. NH_4_^+^ binds to the K^+^ site and enables active transport of Na^+^ and NH_4_^+^. In IMCD, basolateral NH_4_^+^ uptake mediated by the Na^+^,K^+^-ATPase is critical for ammonia and H^+^ secretion, particularly in chronic hypokalemia [115, 117, 119]. Indeed, during hypokalemia, the drop in interstitial K^+^ concentration allows increased NH_4_^+^ binding to the K^+^ site of the Na^+^,K^+^-ATPase, thereby increasing basolateral uptake of NH_4_^+^ [117].

Expression of the Na^+^-K^+^-2Cl^−^ isoform 1 NKCC1 (SLC12A2) has also been observed at the basolateral membrane of A-IC [118]. As with NKCC2 in the TAL, NKCC1 in the ex vivo microperfused rat OMCD can transport NH_4_^+^ by substitution with K^+^. However, its contribution to H^+^ secretion appears to be limited [116]. This observation has been confirmed in *Slc12a2* knockout mice which do not develop any defects in renal acid handling [68, 118].

Studies evaluating the contribution of Rhesus (Rh) glycoproteins as ammonia/ammonium channels have significantly altered our understanding of collecting duct ammonia transport (for review [56, 112, 113, 122, 123]). Two Rh glycoproteins, Rh C glycoprotein (Rhcg, SLC42A3) and Rh B glycoprotein (Rhbg, SLC42A2), have been identified in the renal collecting duct cells. In humans, RhCG appears to be the major ammonia channel, while in mice and rats, Rhbg has been also localized at the basolateral membrane of IC and principal cells [17, 65, 92]. In rodents, Rhcg is present in the apical and basolateral membrane in type A intercalated and principal cells in the distal nephron, including DCT, CNT, CCD, and OMCD. RhCG is detected in the apical plasma membrane of non-A, non-B intercalated cells, but is not present in type B intercalated cells [13, 32, 110]. Rhcg has been identified as a direct NH_3_ channel [7], whereas Rhbg may transport ammonia in the form of NH_3_ and NH_4_^+^ [23]. Rhcg plays a major role in the basolateral ammonia uptake as illustrated by the drastically reduced diffusion of ammonia across the basolateral membrane of ex vivo microperfused CCD from *Rhcg* knockout mice compared to wild type [13]. Both Rhcg and Rhbg expressions are upregulated during chronic metabolic acidosis in mice [98]. However, the importance of Rhbg in t-ammonia transport is more controversial than that of Rhcg [7, 13, 73, 74] as Rhbg knockout mice exhibit either no or a much milder phenotype [5, 6, 25]. Rhbg may have a specific function in the defense against acidosis during hypokalemia [5, 50].

### Carbonic anhydrase and HCO_3_^−^ secretion

In addition to its primary role in bicarbonate production, the carbonic anhydrase II (CAII) also contributes to ammonia secretion in type A intercalated cells. Inhibiting carbonic anhydrase blocks ammonia secretion in isolated perfused OMCD [116]. It is likely that this is because CAII-dependent cytosolic H^+^ production is required to enable parallel H^+^ and NH_3_ across the apical plasma membrane. CAII also facilitates the basolateral efflux of HCO_3_^−^ by activating the Cl^−^/HCO_3_ exchanger AE1 [104]. A*e1 R607H* knockin mouse, which carries the most common dominant dRTA variant of human AE1, R589H, shows a reduced number of type A IC and a decreased expression of B1 subunit of the H^+^-ATPase. One explanation could be that a reduced basolateral anion-exchange activity in type A ICs compromises the traffic and activity of H^+^-ATPase and hence the luminal acid secretion [85]. Another Cl^−^/HCO_3_^−^ exchanger, SLC26A7, also expressed with AE1 at the plasma membrane of type A IC in OMCD, has been involved in the function of type A IC in this segment. *Slc26a7* knockout mice exhibit metabolic acidosis and excrete a more alkaline urine than their wild-type counterparts at baseline [127]. In transfected MDCK, the SLC26A7 abundance increases with medium osmolarity and decreases with extracellular acidic pH [108]. Both AE1 and SLC26A7 could be involved in HCO_3_^−^ reabsorption by type A IC and in the regulation of H^+^ secreting cells in response to modulations in extracellular pH and osmolarity [108]. Their cooperation has not yet been directly demonstrated.

### Luminal secretion

Several in vitro and ex vivo studies have demonstrated that Rhcg is an NH_3_ channel (for review [113]). We were able to show that transepithelial transport of ammonia was drastically reduced in the ex vivo microperfused CCD or OMCD from Rhcg knockout mice due to decreased NH_3_ uptake [7, 13]. A large body of evidence indicates that Rhcg plays a crucial role in ammonia transport with a primary involvement in luminal NH_3_ secretion by collecting duct cells [13]. The first demonstrations were carried out in various mouse models of global or kidney-specific deletion of *Rhcg* gene. In all studies, *Rhcg* knockout mice developed incomplete dRTA with a marked reduction in ammonium excretion in the final urine [7, 13, 74, 75]. Furthermore, Rhcg has been shown to have a crucial role in renal ammonia excretion under different physiological or pathophysiological conditions such as high-protein diet, hypokalemia, ischemia–reperfusion injury, cyclosporine-induced nephropathy, reduced renal mass, or in Cy/ + rats, a model of chronic kidney disease [11, 21, 49, 50, 64, 76]. Membrane expression of Rhcg depends on the amount trafficked to and retrieved from the membrane. Its localization at the plasma membrane is increased by chronic metabolic acidosis and aldosterone [31, 99].

Most ammonia excreted in the final urine comes from secretion from the collecting duct, which requires parallel secretion of NH_3_ and H^+^ to allow the final urine acidification. The multimeric apical plasma membrane H^+^-ATPase protein of the type A IC has been postulated to mediate the majority of H^+^ secretion across the apical plasma membrane of the type-A IC. Mice with a disrupted B1 H^+^-ATPase subunit (ATP6V1B1) develop incomplete dRTA [12, 14, 35, 63]. Comparison of the phenotypes of *Rhcg* and *Atp6v1b1* knockout mice has shown that *Rhcg* knockout mice excrete alkaline urine and little ammonia, whereas *Atp6v1b1* knockout mice have only a mild reduction of their ammonia excretion during a chronic acid load [12, 13]. Cellular studies have established that Rhcg and H^+^-ATPase co-immunoprecipitate in the rat kidney tissue, indicating a close interaction between the two proteins [14]. In CCD from *Rhcg* and *Atp6v1b1* knockout mice, while NH_3_ and H^+^ transports are reduced in CCD from *Rhcg* knockout mice, only H^+^ transport is impaired in CCD from *Atp6v1b1* knockout mice, indicating that Rhcg can modulate H^+^-ATPase activity in CCD cells [14]. This non-reciprocal modulation of the activity of H^+^-ATPase by Rhcg makes sense from a physiological point of view, since NH_3_ is the main buffer of protons in urine.

Electroneutral H^+^,K^+^-ATPase activity has also been reported from the CNT to the IMCD in rats and mice [38]. Under control diet, total H^+^,K^+^-ATPase activity increases during acidosis and potassium deficiency [87, 101]. The role of H^+^,K^+^-ATPase may therefore be more important in potassium deficiency as suggested by a case report of distal renal tubular acidosis with severe hypokalemia in a 21-month-old boy [103]. Two isoforms of the protein are present in the distal nephron: the HK1 or gastric form and the HK2 also known as the “nongastric” or “colonic” H–K-ATPase. Under control diet, both isoforms are contributing to H^+^ secretion in the CCD of mice [79]. Yet, the exact role of H^+^,K^+^-ATPases in ammonia handling is not understood; it has been shown that H^+^,K^+^-ATPases can directly transport NH_4_^+^ but also that their activities can be regulated by ammonia in the renal tissue [37, 107]. However, phenotypic studies of HK1 and HK2 knockout mice subjected to experimental acid loading are lacking to directly establish the role of the H^+^,K^+^-ATPase in the final excretion of ammonia by the kidney.

Other diffusive transporters for NH_3_ movement across the apical and basolateral membranes of the collecting duct may also contribute to ammonia secretion as suggested in cultured mIMCD-3 ([51, 52]). However, this transport is now considered marginal as the deletion of Rhcg in mice abolishes almost 80% of total and apical or basolateral membrane NH_3_ permeability through the CCD or OMCD [7, 13], establishing Rhcg as the main ammonia channel in the collecting duct (Fig. 3).

## Sex differences in ammonia handling by the kidney

The sex difference in ammonia handling by the kidney is an ongoing research topic, which has been studied mainly in mice, so far [22, 81, 124]. A striking sex difference in mouse kidney lies in the structure of the kidney itself, seen both under baseline condition and during acid loading. Male mice have bigger proximal tubules than female mice, and, conversely, the collecting ducts represent a larger volume of the whole kidney in females [53, 54]. On a normal diet, female mice excrete twice as much urinary ammonium as males in urine [54] while having similar food intake and plasma bicarbonate concentration. In addition, female mice show a greater expression of PEPCK, glutamine synthetase, NKCC2, Rhbg, and Rhcg, but not of NBCe1, than male mice [54]. In males, orchidectomy increased urinary ammonium excretion and the expression of PEPCK and NKCC2, both effects being reversed under treatment by testosterone [55]. The authors conclude that the sex difference in proteins involved in ammonia metabolism and transport results in greater ammonia excretion in female than in male mice. We would like to consider an alternative explanation. On steady state in normal mice, a higher ammonia excretion in urine indicates a higher acid production within the body that seems to be androgen dependent. Then, the kidney has to adapt to the higher acid production by producing more ammonia in the proximal tubule and secreting more ammonia along the collecting duct: the different expression of proteins involved in ammonia production and transport could reflect an adaptation to a higher acid production, not being the primary cause of higher ammonia excretion in females.

When subjected to a 7-day HCl loading, both female and male mice were able to carry out a 10- to 15-fold increase in urinary ammonia excretion, indicating that the androgen-dependent difference in ammonia excretion seen on baseline can be eliminated by the need to increase tubular ammonia production and secretion [53]. After 7 days on acid load, no difference in PEPCK, SNAT3, NBCe1, NHE3, Rhbg, and a4 subunit of H^+^-ATPase protein expression could be seen between female and male mice [53, 54]. NKCC2 and Rhcg protein expression remained higher in females than in males [53, 54]. The structural differences between females and males seen on baseline regarding relative cortical proximal tubule and collecting duct volumes were maintained.

The sex difference in kidney structure, specific protein expression, and, likely, metabolic acid production does not prevent both female and male mice to maintain a normal acid–base status on baseline; both male and female mice are able to increase ammonium excretion adequately and similarly on large acid load. Obviously, the mechanisms involved in the sex difference of metabolic acid production, as well as those linking androgens to acid–base homeostasis deserve further investigation.

## Open questions and new directions

Thanks to the development of new molecular and cellular tools and the study of knockout animal models, research carried out in recent years has considerably improved our understanding of ammonia handling in the kidney, its role for the maintenance of acid–base homeostasis, as well as compensatory mechanisms allowing to maintain acid excretion in the context of kidney disease. Nevertheless, gaps in our understanding persist, and new questions have been raised by recent discoveries.

Firstly, a molecular cause is not retrieved in all patients with inherited forms of renal tubular acidosis, either proximal or distal [114]. Full genome sequencing, combined with functional studies of the candidate genes listed in this review, mutated in humans or causing renal tubular acidosis in knockout animal models, such as Kir4.2, NHE4, NBCn1, and Rhcg, can be expected to resolve many of these cases and better explain the underlying mechanisms in the near future.

Secondly, the role of determinants of cell polarity, such as FOXI1, and of signaling or scaffolding, such as WDR72, both recently discovered thanks to gene sequencing in patients, is not yet fully understood. Similarly, the list of protagonists of ammonia metabolism and transport inside and outside the mitochondria of the proximal tubule is still incomplete; the precise role of sulfatides, the lack of which is associated with acidosis, is not understood. Further research is needed to decipher the role of these newly discovered protagonists in the process of acid excretion by the kidney.

Thirdly, the description of sex differences in renal physiology and pathophysiology is still in its infancy, and the understanding of these differences is far from complete.

Finally, although the role and impact of acidosis on the development and progression of CKD has long been known, the mechanisms involved in the adverse effects of acidosis during CKD progression remain incompletely deciphered. The injured kidney responds to the decline in urinary ammonium excretion by increasing t-ammonia production in the remaining functional nephrons. This phenomenon was observed, for example, in the remnant kidney of a CKD stage III rat model of reduced renal mass where an increase in the rate of ammonia production was observed in vitro from the rats fed a high-protein diet [97, 124, 125]. However, this compensatory response can lead to high local intrarenal ammonia concentrations which, in the long term, can trigger deleterious mechanisms in the remaining healthy renal tissue. Stimulation of the complement pathway, recruitment of inflammatory cells, and oxidative stress can be enhanced by high ammonium concentration in renal tissue and promote a cascade of events that may eventually lead to tubulointerstitial fibrosis [29]. A direct link between intrarenal ammonium accumulation and kidney fibrosis has yet to be established in humans. Acidosis also promotes systemic and renal production of endocrine factors such as aldosterone, angiotensin II, or endothelin 1 (ET1). ET1, whose two receptors, ETA and ETB are functional in the renal tissue, contributes to maintain normal bicarbonatemia and blood pH via its ETB receptor by activating transporters leading to bicarbonate reabsorption in the proximal tubule and protons excretion in the collecting duct. However, ET1 also promotes renal injury, proteinuria, inflammation, and kidney tissue fibrosis via its ETA receptor. The mechanisms involved in balancing these two antagonistic actions remain to be identified.

## Conclusion

This review summarizes the state of knowledge on NH_3_/NH_4_^+^ production and transport by the kidney. Fundamental discoveries have altered our understanding of the molecular mechanisms of renal ammonia transport in recent decades. Future research in this field is needed to understand the fate of ammonia metabolism in pathophysiological states and to provide insight into the mechanisms of kidney disease.

## Data Availability

No datasets were generated or analyzed during the current study.
